# Preventing and reducing burnout globally: a six continent thematic assessment

**DOI:** 10.1093/rescon/vmag064

**Published:** 2026-06-23

**Authors:** Mark Linzer, Erin E Sullivan, Trung A Nguyen, Jacqueline Hoare, Fiona Herco, Jennifer Holmes, Paula Gabriela Ponce de León Lovatón, Michaella Alexandrou, Jaskanwal D S Sara, Elizabeth Goelz, Vallire Hooper, Jordan McWilliams, Sara Poplau, Emily O’Brien, Carolyn M Porta, Victor M Montori

**Affiliations:** Department of Medicine and Institute for Professional Worklife, Hennepin Healthcare, Minneapolis, MN 55415, United States; Sawyer Business School, Suffolk University, Boston, MA 02108, United States; Board of Directors and Department of Geriatrics, National Geriatric Hospital and Hanoi University, 1A phuong mai Dong Da Ha Noi, Hanoi, 100000, Vietnam; Department of Psychiatry and Mental Health, University of Cape Town, Neuroscience Institute, Groote Schuur Hospital,, Cape Town, 7925, South Africa; Project Director, Asia Pacific, Institute for Healthcare Improvement, Boston, MA 02109, United States; Workforce Safety and Wellbeing, Governance, Strategy and Research. Safer Care, Melbourne, VIC 3000, Australia; Clinica Aviva, Centros de Salud Peruanos, General Antonio Pezet 959, Dpto 1301 San Isidro, Lima, Peru; Department of Cardiology, Minneapolis Heart Institute Foundation, Minneapolis, MN 55407, United States; Interventional Cardiology, Minneapolis Heart Institute Foundation, Minneapolis, MN 55407, United States; Department of Medicine, Hennepin Healthcare, Minneapolis, MN 55415, United States; Nursing Research, Ascension Health System, St. Louis, MO 63134, United States; Department of Medicine, Hennepin Healthcare Research Institute, Minneapolis, MN 55415, United States; Office of Professional Worklife, Hennepin Healthcare Research Institute and Institute for Professional Worklife, Minneapolis, MN 55415, United States; Duke Clinical Research Unit, Duke University Medical Center, Durham, NC 27701, United States; University of Minnesota School of Nursing, University of Minnesota, Minneapolis, MN 55455, United States; Knowledge and Evaluation Research Unit, Mayo Clinic, Rochester, MN 55905, United States

**Keywords:** workforce, turnover, health policy, collaboration, public health, qualitative research

## Abstract

**Background and Aims:**

Burnout affects millions of healthcare workers worldwide, from food service and nurses to physicians and leaders, impairing worklives and threatening health system stability.

**Methods:**

An international collaborative, PREVAIL, was created to reduce burnout globally. Leaders from six continents shared structured insights into the local state of wellness and burnout contributors and mitigators, which were then subjected to a formal thematic (qualitative) analysis to identify common and region-specific themes.

**Results:**

Across continents, findings showed the global state of well-being was in danger, though threats varied by region: long shifts in Africa, heavy workloads in Asia, austerity-prone environments in Europe and financially driven care in North America. Vulnerable worker groups included women, nurses, and primary care. Mitigators included workload and schedule adjustments (Africa), shift policies with fair pay (Asia), leader support and regular well-being assessments (Australia), improved psychosocial services (Europe), organizational wellness support and career development (North America), and trainee support (South America).

**Conclusions:**

A proposed “common pathway” framework explains shared drivers of poor workforce well-being and provides actionable steps for promoting compassionate, supportive environments worldwide.

**Implications/Learning Points:**

Adopting a coordinated international approach may prevent devastating workforce shortages over the comingdecades. Burnout assessment and reduction in the global workforce is a high priority.

## Introduction

Burnout affects millions of healthcare workers (HCWs), from food service to physicians and leaders, impairing work lives and care for millions of patients worldwide. Minoritized workers facing discrimination and stereotyping, hard to reach due to lack of trust and limited resources in health systems, are often most affected [1].

Burnout has been shown to be caused by four system-level factors: “time pressure and the 3 C’s” (chaos, control, and culture) [2]; importantly, burnout occurs when HCWs don’t feel valued or heard [3]. Deficits in workflows and relationships lead to demoralization, turnover, workforce shortages, and lower quality care. When worklives are robust and healthy, HCWs feel supported and better able to provide empathic, high-quality care [4]. Burnout is lower and satisfaction higher when interventions focus on addressing HCW needs. Members of our team have confirmed impact of work system alignment and interventions [5, 6] such as redesigning workflows, building effective teams, implementing wellness infrastructures, and developing leadership behaviors to honor HCWs’ motivation to care.

What is less clear is how burnout in HCWs has been experienced globally. While many suspect burnout is due to the way healthcare is funded or organized in their countries, there is no clear consensus if burnout is a global phenomenon. Dean and colleagues recently discussed moral injury amid a global health workforce crisis [7], with data provided for two continents (North America and Europe), complementing Aiken’s recent study in Europe and the USA [8]. Our work with the Institute for Healthcare Improvement (IHI) on six continents in diverse groups of HCWs found that burnout exists across numerous continents and that transformative quality improvement projects were feasible and resulted in more engaged workers and satisfied patients. Greater retention of less burned out HCWs could save millions of dollars annually [9, 10] while promoting care continuity, teamwork, and camaraderie. Understanding dimensions of burnout across the globe could engage leaders in change management and spark the spread of interventions within each continent via professional meetings and local networks.

What is not known are the specific contributors to burnout which could underly a global strategic effort to improve HCWs’ lives and patient care. A large grant written for this purpose, PREVAIL (PREVenting And reducIng burnout globaLly), fared well in a highly competitive process. Though not funded, partners across six continents remain unified in their dedication to improve burnout globally.

## Objectives

The primary *objectives* of this article are to summarize the known state of worklives among HCWs globally and assess similarities and differences across continents. The secondary objective is to use current experience and evidence to propose evidence-based recommendations for improving worklives. We hypothesized there would be commonalities across continents, but also discernible differences based upon local health system structures.

## Methods

To characterize worklife across six continents, and guided by partner authors, we reviewed references from Vietnam [11–14], South Africa [15–18], Australia [19–22], Greece/Europe [23–28], Peru/South America [29–32], and the United States (USA)/North America [3, 5–7, 33–36]. The appendix lists numerous additional references reviewed for the project.

### Study design

The study adhered generally to the structure of a narrative review, which provides a comprehensive, though not systematic, overview of the literature with theoretical perspectives. We also included elements of an integrative review, which integrates findings from diverse study types, including theoretical and gray literature [37]. Quality of the review was assessed using the SANRA format “Scale for the Assessment of Narrative Review Articles,” [38] which assesses quality of the review allotting 2 points for each of six criteria (possible score of 12): justification, concrete aims, search methods, referencing, scientific reasoning and data presentation. The main weaknesses of the narrative review method (less rigorous search and article inclusion criteria) were addressed, in part, through a formal thematic analysis of the included information from the geographically diverse authors.

### Document selection

For the review, PREVAIL team leaders around the world (experts in the field of HCW burnout) were asked to provide descriptions of the current state of HCW worklife in their setting and to highlight contributors to burnout within their country or continent. Each leader was asked the same questions about (i) the current state of well-being, (ii) burnout contributors and mitigators, and (iii) benefits of cross-national collaboration. Many of their responses emanated from projects performed locally, some published (e.g. reference 20 by Jordan *et al*. from Victoria, Australia), some not. While local literature was searched by convenience (not systematically) for illustrative findings, there was a wide breadth (Supplementary Figure 1, see online supplementary material) not normally available, including doctoral thesis papers and other academic writings totaling over 100 documents.

### Qualitative analysis

To enhance the structured analysis of responses, a hybrid deductive–inductive thematic analysis was conducted. Data were first organized deductively into three a priori domains (current state of wellness, burnout contributors, and burnout mitigators). Within each domain, inductive coding was then undertaken to identify emergent patterns and themes. Analysis followed an iterative process involving data reduction, code development and refinement, and constant comparison across responses. Coding and data management were facilitated using NVivo15. Codes were subsequently grouped into higher-order themes through repeated analytic review [37]. Given the volume of data, the final analytical step included synthesis in tabular form and member-checking results with PREVAIL leaders. Recommendations from PREVAIL leaders to address burnout are offered using these thematically synthesized findings along with key international references within the text and Supplementary Material. Ethical review was not sought from Hennepin Healthcare’s Institutional Review Board (IRB) due to the project’s descriptive nature without primary data collection.

## Results

### SANRA scoring

The strength of the study design was assessed with the SANRA (Scale for the Assessment of Narrative Review Articles) criteria, with 2 points allotted for each of the six criteria. Overall score was 9 (well above mean score of 6 in the original reference [38]), including justification (2 points), clear aims (2 points), literature search (0 points), reference support for statements (2 points), scientific reasoning (1 point), and data presentation (2 points).

### Current state of well-being and burnout across six continents

To address the primary objective of summarizing wellbeing states globally, we first report the commonalities and differences in prevalence across reports from six continents (Table 1), acknowledging the heterogeneity in burnout definitions and measurement limiting direct comparisons. In Australia, the COVID-19 pandemic precipitated severe burnout, with over 70% of 9518 HCWs reporting moderate to severe symptoms, exacerbated among younger, female, and those with pre-existing mental health conditions [19–22]. Similarly, European HCWs in Greece experienced emotional exhaustion and depersonalization during successive pandemic waves, linked to unsafe working conditions and personnel shortages [39]. South America’s healthcare workforce, exemplified by Peru, displayed variable burnout rates, with urban centers experiencing higher emotional exhaustion, anxiety, and PTSD, reflecting localized pandemic pressures [29, 30, 32]. In the US, burnout peaked above 60% in 2021 [40] but has recently declined, though high stress persists among nurses and primary care providers; this has been shown by others to impact workforce retention and patient safety [33, 36]. Multiple burnout scales were used in these studies, ranging from the Mini Z burnout metric to the Maslach Burnout Inventory, Nursing Stress Scale and Leiden Quality of Work questionnaire to report the prevalence of burnout and severity across each continent (see Table 1).

**Table 1:** The state of wellness across six continents.

Continent	Burnout prevalence and severity	Key affected groups	Major contributing factors	Unique regional characteristics	Impact on healthcare system
Australia	Approximately 70.9% moderate to severe burnout during COVID-19	Young, female workers, those with pre-existing mental health conditions	High pandemic stress, healthcare, and social assistance were described as high-risk sectors	Large economic costs due to unhealthy workplaces	High workplace injury claims; worker health a significant financial burden on organizations
Europe (Greece)	Burnout rates between 46% and 65% depending on role and pandemic wave	Frontline healthcare staff, oncology professionals	Challenges from unsafe hospital environments, staff shortages, reduced leave	Burnout worsened across successive pandemic waves; both public and private sectors affected	Emotional exhaustion and depersonalization prevalent; varying sectors were impacted
South America (Peru)	Burnout ranged from 15% to 45%, higher in urban centers	Physicians, nurses	Job dissatisfaction, fear of COVID-19 contagion	Urban areas experienced elevated burnout and associated anxiety/post-traumatic stress	Burnout linked to fear and job dissatisfaction; significant mental health burdens accompanied burnout
North America (US)	Burnout in physicians peaked near 60% in 2021, currently declining	Nurses, women, primary care providers	High workload and pandemic stress, staffing shortages, salary concerns	Post-pandemic improvement, yet ongoing nursing shortages and retention challenges	High turnover costs affect patient care quality and hospital finances
Africa (South Africa)	Burnout rates ranging from 58.9% to 78% post-pandemic	Junior doctors, general practitioners	Long shifts (up to 30 hours), mental health deterioration, hiring freezes	Worsening mental wellbeing; many healthcare workers are considering leaving profession or country	Severe workforce shortages and increased risk of medical errors
Asia (Vietnam)	Severe burnout widespread	Younger staff and nurses	Heavy workloads, limited control over work	Burnout contributes to increased medical errors and long patient wait times	Efficiency and safety compromised; retention of trained staff challenging

In South Africa, burnout remains alarmingly high, driven by protracted shifts (up to 30 hours), deteriorating mental well-being, and employment insecurity, with many professionals considering leaving the workforce [15–18]. In Asia, exemplified by Vietnam, occupational burnout remains prevalent, particularly among nurses, with emotional exhaustion and heavy workloads [11–14]. Despite shared manifestations globally such as exhaustion and depersonalization, regional differences in economic impact, health system capacity, and workforce policies shape the unique burnout experience, underscoring the need for locally tailored interventions to safeguard HCW well-being globally.

### Factors contributing to burnout

To further contribute to achieving the primary objective, we then looked across all six continents to determine several common factors contributing to burnout, such as excessive workload, staffing shortages, and moral distress; we also found clear differences shaped by local healthcare systems, cultural norms, and economic conditions (Tables 2 and 3).

**Table 2:** Burnout contributors across six continents.

Continent	Distinct burnout factors
Africa	Effects of abrupt foreign aid withdrawal (e.g., USAID in Southern Africa), harsh hierarchical culture, drastic budget cuts creating loss of jobs/moral injury
Asia	High task overload due to low doctor-to-patient ratios (e.g., Vietnam), frequent multi-role expectations, absence of mental health support programs, salary/ resources issues
Europe	Widespread consequences from austerity policies, high emigration (especially young clinicians), fragmented systems with inconsistent psychological support, contract instability (reliance on short term, temporary, or otherwise insecure employment contracts that create ongoing job uncertainty for staff)
North America	Burnout linked to profit-driven healthcare environment, disparities in care access, and issues such as violent/abusive workplaces influencing intention to leave
South America	Broad increases in burnout among nursing professionals, strong statistical link between workload and all burnout dimensions, with variations across subgroups during pandemic surges
Australia	Australian healthcare features unpredictable rostering (schedules), changes in roles, and unique national challenges related to insufficient training and support structures

**Table 3:** Comparison of burnout factor presence across six continents.

Factor	Africa	Asia	Europe	North America	South America	Australia
Staffing shortages	Yes	Yes	Yes	Yes	Yes	Yes
Excessive workload	Yes	Yes	Yes	Yes	Yes	Yes
Resource/funding constraints	Yes	Yes	Yes	Yes	Partial	Yes
Moral distress/injury	Yes	Yes	Yes	Yes	Yes	Yes
Rigid/hierarchical cultures	Yes	Partial	Yes	Yes	Partial	Yes
Mental health support lacking	Yes	Yes	Partial	Partial	Partial	Partial
Unique/national factors	Aid cuts	Multi-role overload	Youth emigration	For-profit focus	Nursing surge	Unpredictable rostering

#### Common factors and identified differences across continents

- Workload and Staffing Shortages: Chronic understaffing and intense workloads are consistent burnout drivers worldwide, whether in Europe’s critical care units [ 25 ], North America’s hospital systems [ 36 ], South America’s nursing workforce [ 30 ], or Africa and Asia’s health facilities [ 11–13, 16, 17].
- Moral Distress and Injury: Across all regions, HCWs report moral distress from being unable to deliver care in accordance with ethical values, often due to resource constraints, external policy decisions, and organizational demands [ 7, 15, 41].
- Resource Constraints: Shortages of supplies and underfunded  systems,  exacerbated by budget cuts or abrupt ends of international funding, notably in South Africa [42], directly affected staff well-being and led to burnout.
- Organizational and Leadership Issues: Non-supportive cultures, rigid hierarchies, lack of autonomy, poor communication, and unstable contracts (short term or otherwise insecure employment arrangements) were frequent contributors to burnout globally [ 16, 20, 23, 29].
- COVID-19 Pandemic Impact: The pandemic intensified pre-existing challenges widely, causing spikes in burnout rates and worsening work conditions, mental health, and job security [ 13, 16, 19, 24, 30–32].

### Mitigation strategies

For our secondary objective, we then sought utilized mitigation strategies. While each continent tailored their approaches to local challenges, several *shared themes emerged globally* (Table 4). These included organizational policy reforms, improved mental health support, better working conditions, and commitments from leadership.

**Table 4:** Burnout mitigation strategies by continent.

Continent	Key strategies	Notable programs and features
Africa	Organization-level interventions (modifying workloads, breaks, schedules), autonomy, supervisor support, wellness training	Retaining senior staff, mentor programs, resilience building, peer team initiatives
Asia	Shift rotation policies, digital health solutions, fair pay, leadership investment, tailored stress management, peer support, and mindfulness	Mental Health Care Handbook for Healthcare Workers, workshops, self-assessment, resilience/leadership development efforts
Australia	“Joy in Work” (IHI) framework, team huddles, addressing frustrations, flexible rostering, regular wellbeing assessment, leadership support	Emphasis on affordable/systemic change, common wellness language, permission to prioritize wellness
Europe	Universal access to mental health care, anti-stigma actions, psychosocial services, monitoring framework, staff recognition	WHO and EU programs, cross-national projects, multi-level burnout interventions
North America	Guidance from US Surgeon General, well-being frameworks, Chief Wellness Officers, community/cohesion, work-life balance, opportunity for growth	Advocacy for policy action, peer sharing, leadership emphasis on sustainability
South America	Monitoring shift durations, communication training, support for trainees, counselling, prevention networks, and embedding these supports into organizational structures so they are sustained over time	RESPIRA program (Peru) to support COVID patients’ care, extended support to trainees, integrating research and training

EU: European Union; IHI: Institute for Healthcare Improvement; USA: United States of America; WHO: World Health Organization.

#### Shared mitigation strategies across continents

Most continents emphasized proactive well-being policies, regular burnout measurement, mental health support, peer networks, communication training, flexible scheduling, and leadership engagement. Many regions highlight system- rather than individual-level change.

#### Cross-continent themes

- Leadership Buy-In and Worker Engagement: Across continents, leadership commitment was crucial for success; well-being initiatives work best when supported by top leaders and when frontline  staff are  involved in changes.
- Regular Assessment and Monitoring: Most regions recommend regular measurements of burnout and staff well-being to adjust strategies and track progress [ 20, 35, 39].
- Tailored Mental Health Support: Both Asia and Europe developed targeted mental health handbooks and intervention toolkits, often including self-assessment [ 43–45 ].
- Shift and Workload Management: Monitoring shift lengths and ensuring fair scheduling were most prominently noted in South America [ 46 ].
- Peer Networks and Mentoring: Australia highlighted mentorship, team support, and occupational violence prevention, with Africa also employing these interventions while additionally leveraging resilience-building in trainees [ 20, 47, 48].
- Education and Communication Skills: Many regions used training in communication and coping with uncertainty to build staff confidence and reduce emotional exhaustion.

These strategies demonstrated the importance of organizational culture, routine measurement, leadership involvement, mental health resources, fair workload, and peer support in reducing burnout for HCWs globally, and relate to a “common pathway” for action highlighted below.

### Benefits of international collaboration

Although not subjected to formal qualitative assessment, comments from PREVAIL leaders echoed a common appreciation of sharing experiences and working collaboratively. They felt by participating in global initiatives, they could learn best practices, standardize stress-reduction programs, learn means to increase peer-support programs, highlight locally successful policy reforms, recognize their shared humanity, and have local teams benefit from international expertise that could lead to new practices, reduce duplication, and increase pace of scaling. Underlying this was a sense that “no country is immune” to burnout, and that mutual collaboration will be required to extinguish it.

## Discussion

Our findings suggest a “common pathway” that results from health systems and governments withholding resources needed for patients and clinicians to engage in the moral work of care. Respondents characterized geographically diverse ways in which resource deprivation is enacted: poorly designed workflows resulting in a loss of precious HCW mental resources, poverty faced by millions of patients globally, and management inattention to well-being leave many clinicians with too much work and too few resources. Sustained austerity policies without changing the ways healthcare is organized exacerbate these situations. The extraction of value from necessary and desirable care leaves few resources (Supplementary Figure 2, see online supplementary material). These effects can be direct or indirect.

Directly, by not having enough resources, clinicians must often move patients mechanically through care, an exercise in matching anticipated patient needs with a test, treatment, or referral. This leaves clinicians frustrated, not having time to notice subtle aspects of illness, discover what contributes to a patient’s situation, or co-create meaningful care plans. Without resources, teamwork is impaired, care becomes hurried (“blurred”), and clinicians and patients are unable to adapt work conditions to work they should be doing [49]. As the diverse data sources integrated here indicate, these factors negatively affect clinician well-being.

Indirectly, clinicians experience constrained conditions for care. This may reflect policy decision makers placing a relatively lower value on caring itself [1, 15, 29, 35]. This may erode HCWs’ sense of self-worth or perceived value [35]. The work conditions can reduce empowerment and induce moral injury (going against one’s ethical beliefs).

We speculate that a “common pathway” framework may explain why clinician wellbeing suffers globally, despite large differences in financing, organization, and practices of health systems. If correct*, this framework would predict healthier clinical workforces if actions were taken* to prioritize investment in areas that have constrained health systems, allowing workers to gain sufficient control over workloads (control being a known burnout mitigator, Supplementary Figure 2, see online supplementary material).

To the extent that this postulated pathway is common, we would expect that remedies could have global effectiveness. Such remedies would confront the evident reality that *access and other aspects of healthcare cannot be improved at the expense of the conditions that make caring possible, including the well-being of the workforce.* Reducing waste and constructing healthcare budgets and policies that address social determinants of health, invigorate public health, and promote care skills of workers would contribute to rightsizing resources while reducing demands for addressing suffering. Health system leaders can explicitly value clinicians’ work (feeling valued being another known burnout mitigator, 3) and give clinicians more discretion over work conditions [49], including time for supportive teamwork and unhurried workflows. The common pathway framework (Supplementary Figure 2, see online supplementary material), with burnout, quality and safety as primary dependent variables and adverse work conditions as independent predictors, also implies that treating unwell clinicians in isolation or focusing on improving resilience [50] cannot induce durable benefits on well-being.

These findings suggest actionable interventions to reverse a trend of high burnout and suboptimal HCW well-being globally. Subtle differences are noted across continents, with burnout contributors varying from long shifts and heavy workloads in Africa [16] and Asia [12–14] to austerity-driven environments and unsafe workplaces in Europe [25, 26, 28], and financially driven care in the United States [34, 35]. There was a commonality in the identified *vulnerable groups, which consistently included young women, nurses, and those in primary care, justifying focused interventions in these groups*. There were successful, locally focused mitigators, including organization-level interventions on schedules in Africa, shift policies with fair pay in Asia [51], huddles, leader support, and regular well-being assessments in Australia [20], mental health access and psychosocial services in Europe [23], organizational well-being leaders and career development in the USA [35, 36], and capped shift durations and support for trainees in South America [30, 46]. This list is a reminder that *change has been accomplished, and that scaling of changes could be impactful*. A missed opportunity seen in our findings is *the infrequent identification of patients’ views of these difficult circumstances*. We believe a *partnership with patients* could bring greater attention to this multi-national burnout crisis.

Turning to the global, continent-based findings and their implications, as sought in our primary objective, Table 1 paints a challenging picture of the burnout landscape. Prevalence worsened during the pandemic. Rates were generally high, averaging 40%–65%, albeit with varying definitions of burnout. As noted, groups at high risk (young women) were consistent across continents [10, 14, 19, 26, 29, 30]. The data also reflected unsafe work environments, high workloads, staff shortages, and lack of work control [12–14, 15, 21, 26, 29], with a risk of medical errors and staff mental illness. Exhaustion was prevalent, and turnover costs affected fiscal resources. To our knowledge, this is the first time the serious consequences arising from burnout have been articulated globally.

Tables 2 and 3 highlight burnout contributors. While the final result of the burnout experience appears relatively consistent, the means of arriving there likely vary by location. Thus, in Africa, decreases in foreign aid with a decline in investment in infrastructure and longstanding hierarchical cultures led to work stress [15, 16], while the Asian experience focused on work overload and an absence of mental health support [12]. Europe has been challenged by austerity programs and emigrating young physicians [8, 52], the United States by profit-driven healthcare and workplace violence [33, 36], South America and the USA by nursing workload challenges [30, 33], and Australia by lack of predictable work and insufficient training [19]. These data not only provide a roadmap for each continent, but indicators that may apply across continents, as their presence can herald the onset of work challenges and widespread burnout.

Table 4 highlights effective mitigation strategies. Not only are the depth and breadth encouraging, but the observed consequences of employing them suggest a brighter future for eliminating burnout. In Africa, workload modifications, breaks, greater autonomy, supervisor support, and well-being training contributed to retention, more mentoring, HCW resilience, and peer support programs. Asia described shift policies, fair pay, and stress management programs resulting in mental health handbooks. Australia institutionalized “joy in work” programs, team huddles, flexible rostering, well-being assessments, and leader support [20], and found affordable system changes, a common well-being language, and more frequent permission to prioritize well-being. In Europe, programs provided access to mental health support and focused on reducing stigma and recognizing staff’s accomplishments [23]; outcomes included cross-national projects and multilevel burnout interventions. In the USA, federal support [34], a rapid rise in Chief Wellness Officer positions, and career growth opportunities fed public advocacy and programs for leader and worker sustainability, while South America monitored shift durations, provided trainee support and counseling, and saw greater integration of research and training and a better spirit among trainees. These global programs suggest that despite the dire state of HCW well-being after the pandemic, evidence-driven efforts to improve well-being can be effectively instituted. In line with our secondary objective, we provide a list of discrete recommendations for action in Table 5.

**Table 5:** Recommendations to reduce burnout globally.

Areas of suggested focus	Comments
Leadership	
Supported	Avoid leader burnout
Trained in wellbeing	Teach leaders how to do this
Empathetic	Empathy training
Safety	
Psychological	Foster environments where workers can speak up
Physical	Reduce risks of workplace violence
Wellbeing measurement	Standardize use of specific measures
Work flexibility	Important for those with work and home activities
Teamwork	Use teamwork toolkits
Peer support programs	Allot resources to train peer supporters
Policy changes—wellbeing lens	View workplace changes through a wellness lens
Manageable, predictable workloads	Constantly monitor and make sure workload is doable
Resilience/self-care	Make these available, but not the sole focus
Work–life balance	Explicitly supported
Moral injury reduction	Define and prevent it
Promote feeling valued	Use evidence-based methods
Transparent communication	Frequent, honest, open
Positive organizational culture	Reduce hierarchies; promote trust
Minimize impact of role changes	Train for redeployment
Restore financial balance and equity	Use cost-effective interventions; reduce turnover

Inspired by table in Smallwood, *BMJ Leader* (19).

The main lesson learned from this “30 000-foot view” is that burnout in healthcare is a global phenomenon, and it will take global cooperation and many organizations working together to standardize (using regular measurement and system level supports) and then customize approaches (with data-driven, locally implemented interventions) that can turn this crisis around. This builds upon recent literature [53–56], which calls attention to the challenges [57], and augments the literature by capturing the scope and nuances of global burnout with tailored recommendations for its elimination.

There are limitations and strengths to this analysis. Data were provided by thought leaders and workers within one country on each continent, and while asked to reflect on the entire continent’s experience, the data mainly represent the experience of the country, and in cases sub-regions, of the writer(s) and their positionality with respect to the subject matter. There were no formal inclusion or exclusion criteria for selection of references, as this was not a systematic review; this may limit the applicability of our findings to particular jurisdictions. However, there was a wide breadth of sources, including doctoral theses and other less often cited “grey literature” documents; these sources add to the richness and texture of the findings. Burnout measures varied by continent, with predictable heterogeneity of numerical findings (such as burnout prevalence). Finally, due to the analysis being qualitative, we were careful not to infer effect sizes or magnitude of effects and were unable to calculate confidence intervals or interquartile ranges. The analysis, however, was formally structured and thematically driven, allowing production of a multi-layered, thematic array of challenges and successes to explain the present while guiding potential activities to ensure a better future.

The future of global burnout reduction will require communication, partnerships, funding, and support. Global conferences, such as the annual WELLMED meeting in Greece, or the bi-annual International Conference on Physician Health, coupled with continental spread through regional meetings, will keep health systems informed on current, evidence-based approaches. Regular measurement of work conditions and worker reactions, as well as the advent of affordable wearable devices that detect, monitor and respond to stress, fatigue, and psychological strain through objective biological signals, can increase workplaces’ ability to respond to real-time indications of HCW distress. There also is a need to identify the role for *patients as partners*, bringing their power as collaborators in improving worklife in their care providers.

Table 5 portrays viable options for change, including a guided approach to improving leader support, worker and patient safety, organizational cultures for care, well-being measurement, work flexibility, strong teams, a well-being lens for organizational changes, workload monitoring, efforts to promote workers’ feeling valued, transparent communication, and sufficient financial support for workers in need. These emphases are likely to improve well-being in the workforce and can be applied widely (many without great expense) to restore trust and commitment within the healthcare workplace.

Our logic model for international collaborative programs (Supplementary Figure 3, see online supplementary material for a color version of this figure) provides a framework for global intervention, from Resources (staff/partner organizations/Improvement Advisors) to Activities (well-being champions and toolkits), Outputs (HCWs reached, patients involved in system change, organizations changed), Outcomes (local infrastructures, improved well-being, international networks) and Impact (lower burnout by a relative 50% by 2030, with warmer care, improved retention, and costs neutral or reduced from lower turnover).

### Implications

Burnout appears to be widespread across six continents. We propose that a cohesive program for burnout reduction will require sites engaging with major funding initiatives to guide global study and implementation. Future research studies should focus efforts on identifying and estimating the effect on burnout of aggravators and mitigators, including varied impacts on those of different genders, ethnicities, roles and early vs late career subgroups; these findings could guide local implementation efforts. While the PREVAIL team will continue to watch for opportunities to launch this initiative, we encourage local, national, and continent-wide groups to advance this work, for the sake of our workers, our patients, and our planet.

## Supplementary Material

vmag064_Supplementary_Data

## Data Availability

Because there are no primary data, there are no data to share.
