# Supplementary material for: Preventing and reducing burnout globally: a six continent thematic assessment
Source: Res Connect. 2026 Jun 23;1(2):vmag064. doi: 10.1093/rescon/vmag064 (PMC13290463; doi:10.1093/rescon/vmag064)
Supplement: vmag064_Supplementary_Data [file vmag064_supplementary_data.zip › Appendix Figure Legend.docx]

Appendix Figure Legend

Appendix Figure 1

Supplemental References

Appendix Figure 2

Common pathway framework of factors (inputs) causing and consequences (intermediate and final outcomes) of burnout among healthcare workers globally.

Appendix Figure 3

Logic model demonstrating means of structuring and assessing impact of international collaborative programs focused on burnout reduction. HCW = healthcare workers.
