# Supplementary material for: Preventing and reducing burnout globally: a six continent thematic assessment
Source: Res Connect. 2026 Jun 23;1(2):vmag064. doi: 10.1093/rescon/vmag064 (PMC13290463; doi:10.1093/rescon/vmag064)
Supplement: vmag064_Supplementary_Data [file vmag064_supplementary_data.zip › Appendix Figure 1.docx]

South Africa

1S. The state of health in South Africa: Reflections and future directions | SAMRC. Accessed March 13, 2025. https://www.samrc.ac.za/news/state-health-south-africa-reflections-and-future-directions

2S. MPS RSA. https://www.medicalprotection.org/southafrica/casebook-and-resources/medicolegal-articles-and-features/view/survey-shines-a-light-on-mental-wellbeing-of-doctors-in-south-africa

3S. The struggles of junior doctors in South Africa. Accessed March 13, 2025. https://www.iol.co.za/thepost/opinion/the-struggles-of-junior-doctors-in-south-africa-eb203626-2db8-4814-8542-68ad6d8eca60

4S. Mendelson M, Boloko L, Boutall A, et al. Clinical management of COVID-19: Experiences of the COVID-19 epidemic from Groote Schuur Hospital, Cape Town, South Africa. S Afr Med J. 2020;110(10):973-981. doi:10.7196/samj.2020.v110i10.15157

5S. Mendelson M, Booyens L, Boutall A, et al. The mechanics of setting up a COVID-19 response: Experiences of the COVID-19 epidemic from Groote Schuur Hospital, Cape Town, South Africa. S Afr Med J. 2020;110(10):968-972. doi:10.7196/samj.2020.v110i10.15215

6S. Hoare J, Frenkel L. South Africa. Lancet Psychiatry. 2021;8(10):865. doi:10.1016/s2215-0366(21)00365-5.

Vietnam

7S. Nguyen MQ, Le PAH, Vo MHN. A Study on The Relationship Between Burnout and Quality of Life Among Nurses In Thu Duc District Hospital, Vietnam. Published online June 11, 2020. doi:10.21203/rs.3.rs-33664/v1

8S. Tran B, Nguyen MT, Auquier P, et al. Psychological impacts of COVID-19 on Vietnamese health workers over the prolonged restricted COVID-19 responses: a cross-sectional study. BMJ Open. 2023;13(8):e069239. doi:10.1136/bmjopen-2022-069239

9S. General Statistics Office Yearbook of Vietnam 2023. Statistical Publishing House; 2024.

10S. Pham TNT, Pham TTH, Nguyen BT. The situation of occupational stress of original surgery of obstetrics and gynecology and some related factors. Journal of Nursing Science. 2023;6(02):141-152. doi:10.54436/jns.2023.02.570

11S. Hanh LTK, Toan NV, Hai VM, Anh TQ. Factors associated with stress among healthcare workers at Hanoi Medical University Hospital and Thai Binh Medical University Hospital. TCNCYH. 2023;167(6):253-262. doi:10.52852/tcncyh.v167i6.1637.

12S. Nguyen VHA, Phan YTH, Vuong TNT, et al. The Relationship Between Burnout, Stress, And Resilience Among Vietnamese Health Care Workers. National Journal of Community Medicine. 2024;15(03):215-226. doi:10.55489/njcm.150320243557

13S. Ministry of Health. Guidelines on Ensuring Occupational Safety and Hygiene for Healthcare Workers in COVID-19 Prevention and Control, issued under Decision No. 838/QĐ-BYT. 2022.

14S. Ministry of Health. Digital Health Strategy toward 2025 and Vision to 2030. 2020.

15S. Hung Vuong Hospital. Staff of Hung Vuong Hospital overcome “burnout syndrome” caused by the COVID-19 pandemic. Hung Vuong Hospital. Accessed March 26, 2025. https://bvhungvuong.vn:443/tin-tuc/nhan-vien-benh-vien-hung-vuong-chien-thang-hoi-chung-burnedout-do-dai-dich-covid19-gay-ra.

16S. Watanabe K, Tran TTT, Sripo N, et al. Effectiveness of a Smartphone-Based Stress Management Program for Depression in Hospital Nurses During COVID-19 in Vietnam and Thailand: 2-Arm Parallel-Group Randomized Controlled Trial. J Med Internet Res. 2024;26:e50071. doi:10.2196/50071.

Europe:

17S. Karlafti E, Benioudakis ES, Barouxi E, Kaiafa G, Didangelos T, Fountoulakis KN, et al. Exhaustion and burnout in the healthcare system in Greece: A cross-sectional study among internists during the COVID-19 lockdown. Psychiatriki. 2022;33:21-30.

18S. Pittaka M, Sakellakis M, Metaxas V, Kardamakis D, Wagland R. Burnout Syndrome among Doctors in Greek Oncology Departments. Iran J Psychiatry. 2022;17:162-76.

19S. Sikaras C, Pachi A, Alikanioti S, Ilias I, Sideri EP, Tselebis A, et al. Occupational Burnout and Insomnia in Relation to Psychological Resilience Among Greek Nurses in the Post-Pandemic Era. Behavioral Sciences. 2025;15:126.

20S. Alexias G, Papandreopoulou M, Togas C. Work Engagement and Burnout in a Private Healthcare Unit in Greece. Int J Environ Res Public Health. 2024;21.

21S. Koutsouri AK, Gkentzi D, Paraskevas T, Michailides C, Papantoniou K, Kavvousanos M, et al. Burnout Among Healthcare Workers During Covid-19 Pandemic: Results from Seven Hospitals in Western Greece. Mater Sociomed. 2023;35:285-9.

22S. Rachiotis G, Kourousis C, Kamilaraki M, Symvoulakis EK, Dounias G, Hadjichristodoulou C. Medical supplies shortages and burnout among Greek health care workers during economic crisis: a pilot study. Int J Med Sci. 2014;11:442-7.

23S. Bazoukis X, Kalampokis N, Papoudou-Bai A, Bazoukis G, Grivas N. The increasing incidence of immigration and information-seeking behaviour of medical doctors in north-western Greece. Rural Remote Health. 2020;20:4877.

24S. Sikioti T, Zartaloudi A, Pappa D, Mangoulia P, Fradelos EC, Kourti FE, et al. Stress and burnout among Greek critical care nurses during the COVID-19 pandemic. AIMS Public Health. 2023;10:755-74.

25S. Vlassi A, Vitkos E, Michailidou D, Lykoudis PM, Kioroglou L, Kyrgidis A, et al. Stress, Professional Burnout, and Employee Efficiency in the Greek National Organization for the Provision of Health Services. Clinics and Practice. 2023;13:1541-8.

26S. EPAPSY. Psychosocial Support Program “P-Support”: EPAPSY; 2020 [Available from: <https://www.epapsy.gr/en/psychosocial-support-program-p-support/>.

27S. Ekathimerini. Niarchos Foundation offers bonus to hospital staff 2020 [Available from: <https://www.ekathimerini.com/news/252205/niarchos-foundation-offers-bonus-to-hospital-staff/>.

28S. Organization WH. Greece takes steps to improve mental health at work using WHO guidance: Copenhagen: WHO Regional Office for Europe; 2024 [Available from: <https://www.who.int/europe/news/item/10-10-2024-greece-takes-steps-to-improve-mental-health-at-work--using-who-guidance>.

29S. National Action Plan for Mental Health 2021–2030: 10 intervention axes for the protection of the citizens’ mental health and the improvement of the mental health services provided in Greece Athens: Ministry of Health; 2023 [Available from: <https://www.moh.gov.gr/articles/health/domes-kai-draseis-gia-thn-ygeia/c312-psyxikh-ygeia/c685-draseis/11499-ethniko-sxedio-drashs-gia-thn-psyxikh-ygeia>.

30S. (EU-OSHA) EAfSaHaW. OSH Pulse 2022: Workers’ mental health after the pandemic [Available from: <https://osha.europa.eu/en/publications/osh-pulse-2022-workers-mental-health-after-pandemic>.

31S. (ETUI) ETUI. New report on how European countries address the issue of burnout in the workplace Brussels in 2022 [Available from: <https://www.etui.org/topics/health-safety-working-conditions/news-list/new-report-on-how-european-countries-address-the-issue-of-burnout-in-the-workplace>.

32S. Consortium H-W. H-WORK Project: Multilevel interventions to promote mental health in the workplace: Horizon 2020/Horizon Europe; 2020 [Available from: <https://h-work.eu/project/>.

33S. Consortium M. Mental Health Promotion and Intervention in Occupational Settings 2020 [Available from: <https://www.mentuppproject.eu/project/>.

USA:

34S. Gunja, M.Z., Gumas, E.D, Williams, R.D. US Health Care from a Global Perspective, 2022: Accelerating Spending, Worsening Outcomes. doi:10.26099/8ejy-yc74.

35S. Valaitis K. Exploring the US Healthcare System. University of West Florida Pressbooks; 2023. Accessed September 24, 2025. <https://pressbooks.uwf.edu/ushealthcaresystem>.

36S. Trends in healthcare spending. American Medical Association. 2025. Accessed September 24, 2025. <https://www.ama-assn.org/about/ama-research/trends-health-care-spending>.

37S. Nursing Solutions. NSI National_Healthcare_Retention_Report; 2025. Accessed April 8, 2025. <https://www.nsinursingsolutions.com/Documents/Library/NSI_National_Health_Care_Retention_Report.pdf>.

38S. World Health Organization. State of the World’s Nursing 2025: Investing in Education, Jobs, Leadership and Service Delivery. 1st ed. World Health Organization; 2025.

39S. Smiley RA, Kaminski-Ozturk N, Reid M, et al. The 2024 National Nursing Workforce Survey. J Nurs Regul. 2025;16(1):S1-S88. doi:10.1016/S2155-8256(25)00047-X.

40S. Patrician PA, Bakerjian D, Billings R, et al. Nurse well-being: A concept analysis. Nurs Outlook. 2022;70(4):639-650. doi:10.1016/j.outlook.2022.03.014.

41S. Stamm BH. The Concise ProQOL Manual. 2nd ed.; 2010. Accessed July 26, 2024. [www.ProQOL.org](http://www.ProQOL.org).

42S. Barré JH, Hooper V. An Integrative Review of Measures of Secondary Traumatic Stress. | J Nurs Measurement | EBSCOhost. doi:10.1891/JNM-2021-0045

43S. Rushton CH. Cultivating Moral Resilience. Amer J Nurs. 2017;117(2):S11. doi:10.1097/01.NAJ.0000512205.93596.00.

Peru references in ENGLISH:

44S. Muñoz del-Carpio Toia A, and Gonzales R. Burnout syndrome in healthcare workers at Goyeneche Hospital II in Arequipa, Peru . Medical Horizonte. 2022;22:12–19. <https://scielo.cl>.

45S. Rosales-Saade A. Burnout syndrome in healthcare personnel during the pandemic in a highly specialized hospital (Horizonte Médico). 2025. <https://horizontemedico.usmp.edu.pe>. (accessed online).

46S. UPCH Repository. Burnout syndrome in hospital physicians and nurses: institutional reports / theses (Repository). Cayetano Heredia Peruvian University. <https://repositorio.upch.edu.pe>. (accessed online).

47S. Peruvian Medical Association presents comprehensive support program for physicians: RESPIRA. 2025. Retrieved from https://www.cmp.org.pe (accessed online).

48S. Government of Peru — National Institute of Mental Health. The Peruvian Medical Association launches “Respira”: a program that puts the mental health of physicians at the center of attention. August 15, 2025. Retrieved from https://www.gob.pe. (accessed online).

49S. Alicia – Concytec. Burnout syndrome levels among workers at the Progreso Health Center, Chimbote, April to September 2020 [Bachelor's thesis, San Pedro University]. ALICIA Repository. <https://alicia.concytec.gob.pe/vufind/Record/USPE._2e853ec85831de5b5509c48a7012810d>.

50S. Holguín Riccer, K. Frequency of burnout syndrome among medical interns at a private university in Metropolitan Lima, 2024 [Bachelor's thesis, Universidad Peruana Cayetano Heredia]. UPCH Repository. <https://repositorio.upch.edu.pe/bitstream/handle/20.500.12866/16573/FrecuenciaHolguinRiccerKaterine.pdf>.

51S. Cayetano Heredia Hospital. Burnout syndrome in medical residents in COVID-19 areas of Cayetano Heredia Hospital [Specialty thesis]. Cayetano Heredia Peruvian University. 2020. <https://repositorio.upch.edu.pe/handle/20.500.12866/8617>.

52S. Infobae. Peru is the country with the highest level of burnout in Latin America: 1 in 6 employees suffers from frequent burnout at work. Infobae Peru. May 26, 2025. <https://www.infobae.com/peru/2025/05/26/peru-es-el-pais-con-mas-burnout-laboral-en-latinoamerica-1-de-cada-6-empleados-sufre-agotamiento-frecuente-en-su-trabajo>.

53S. Pérez Rojas L. Burnout syndrome in healthcare professionals at an intermediate care hospital in Metropolitan Lima. Ciencia Latina Multidisciplinary Scientific Journal. 2024;8(2):493–506. <https://ciencialatina.org/index.php/cienciala/article/view/18043>.

54S. Ruiz L and García P. Burnout syndrome in healthcare personnel at San Juan de Dios Hospital during the COVID-19 pandemic [Bachelor's thesis, Universidad Católica Sedes Sapientiae]. RENATI. 2021. <https://renati.sunedu.gob.pe/handle/renati/414454>.

55S. César Vallejo University. Level of burnout syndrome in nurses at San Juan de Lurigancho Hospital, Lima, Peru. 2021 [Bachelor's thesis, UCV]. UCV Repository. <https://repositorio.ucv.edu.pe/handle/20.500.12692/91435>.

56S. Federico Villarreal National University. Burnout syndrome in human medicine students at UNFV, Lima 2024. [Bachelor's thesis]. UNFV Repository. https://repositorio.unfv.edu.pe/handle/20.500.13084/10306.

57S. Cayetano Heredia Peruvian University. Frequency of burnout syndrome and fear of COVID-19 among frontline healthcare workers in Lima, Ica, and Tacna, 2021. [Research thesis]. UPCH Repository. https://repositorio.upch.edu.pe/handle/20.500.12866/11703.

58S. Peruvian University of Applied Sciences. Cumulative incidence of burnout syndrome in university students: longitudinal follow-up 2021-2023. Neuropsychiatry J. UPCH. 2024;87(3):211-20. https://revistas.upch.edu.pe/index.php/RNP/article/view/5269.

59S. Cassaretto Bardales M, Chau Pérez Aranibar C, Espinoza Reyes M del C, Otiniano Campos F, Rodríguez Cuadros L and Rubina Espinosa M. Mental health in university students of Consortium of Universities during the pandemic. Consortium of Universities. 2021. <https://www.consorcio.edu.pe/wp-content/uploads/2021/10/SALUD-MENTAL-CONSORCIO-DE-UNIVERSIDADES.pdf>.
