# Supplementary figures and images for: Preventing and reducing burnout globally: a six continent thematic assessment
Source: Res Connect. 2026 Jun 23;1(2):vmag064. doi: 10.1093/rescon/vmag064 (PMC13290463; doi:10.1093/rescon/vmag064)

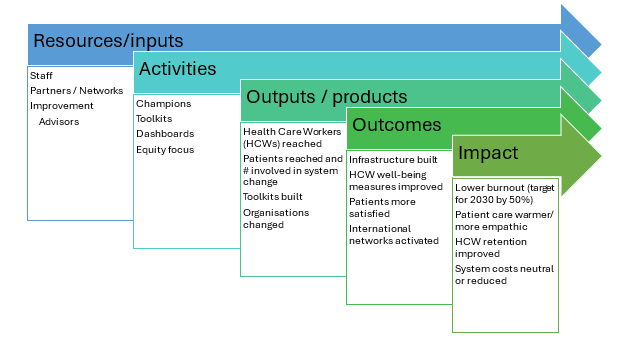

Supplement: vmag064_Supplementary_Data [file vmag064_supplementary_data.zip › Appendix Figure 3 Newer.png]

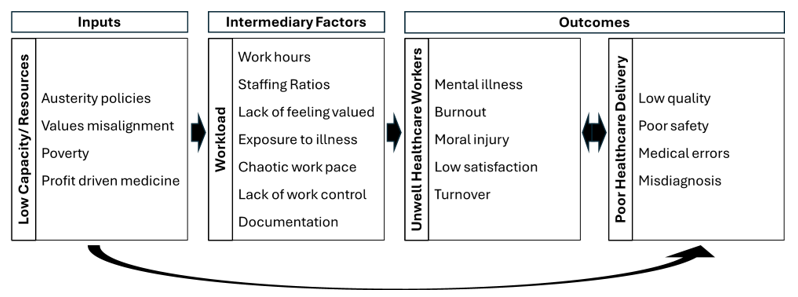

Supplement: vmag064_Supplementary_Data [file vmag064_supplementary_data.zip › Appendix Figure 2 New.png]
